# Experimental infections of different carp strains with the *carp edema virus* (CEV) give insights into the infection biology of the virus and indicate possible solutions to problems caused by koi sleepy disease (KSD) in carp aquaculture

**DOI:** 10.1186/s13567-017-0416-7

**Published:** 2017-02-21

**Authors:** Mikolaj Adamek, Anna Oschilewski, Peter Wohlsein, Verena Jung-Schroers, Felix Teitge, Andy Dawson, David Gela, Veronika Piackova, Martin Kocour, Jerzy Adamek, Sven M. Bergmann, Dieter Steinhagen

**Affiliations:** 1 Fish Disease Research Unit, Institute for Parasitology, University of Veterinary Medicine, Bünteweg 17, 30559 Hannover, Germany; 2 Department of Pathology, University of Veterinary Medicine, Bünteweg 17, 30559 Hannover, Germany; 3 School of Life Sciences, Keele University, Keele, ST5 5BG UK; 4 South Bohemian Research Centre of Aquaculture and Biodiversity of Hydrocenoses, Faculty of Fisheries and Protection of Waters, University of South Bohemia in Ceske Budejovice, Zatisi 728/II, 389 25 Vodnany, Czech Republic; 5 Experimental Fish Farm in Zator, The Stanislaw Sakowicz Inland Fisheries Institute in Olsztyn, 32-640 Zator, Poland; 6 Institute of Infectology, Friedrich-Loeffler-Institut, Südufer 10, 17498 Greifswald-Insel Riems, Germany

## Abstract

Outbreaks of koi sleepy disease (KSD) caused by *carp edema virus* (CEV) may seriously affect populations of farmed common carp, one of the most important fish species for global food production. The present study shows further evidence for the involvement of CEV in outbreaks of KSD among carp and koi populations: in a series of infection experiments, CEV from two different genogroups could be transmitted to several strains of naïve common carp via cohabitation with fish infected with CEV. In recipient fish, clinical signs of KSD were induced. The virus load and viral gene expression results confirm gills as the target organ for CEV replication. Gill explants also allowed for a limited virus replication in vitro. The in vivo infection experiments revealed differences in the virulence of the two CEV genogroups which were associated with infections in koi or in common carp, with higher virulence towards the same fish variety as the donor fish. When the susceptibility of different carp strains to a CEV infection and the development of KSD were experimentally investigated, Amur wild carp showed to be relatively more resistant to the infection and did not develop clinical signs for KSD. However, the resistance could not be related to a higher magnitude of type I IFN responses of affected tissues. Despite not having a mechanistic explanation for the resistance of Amur wild carp to KSD, we recommend using this carp strain in breeding programs to limit potential losses caused by CEV in aquaculture.

**Electronic supplementary material:**

The online version of this article (doi:10.1186/s13567-017-0416-7) contains supplementary material, which is available to authorized users.

## Introduction

Common carp (*Cyprinus carpio* L. or *Cyprinus rubrofuscus* L. in some Asian strains) is one of the most important fish species for aquaculture with an annual production of about 3.8 million metric tons, which accounted for 5.7% of global aquaculture production (including fish, crustaceans and molluscs) in 2012 [1]. Aquaculture of carp provides food for people, mainly in rural areas [2] and their extensive production in shallow ponds is considered environmentally friendly as these ponds also provide natural habitats for many aquatic organisms and are beneficial for regional water balance [3]. In addition, coloured morphotypes of carp, i.e. koi, are popular ornamental fish species [4]. Further development of carp aquaculture and koi trade is challenged by the emergence of infectious diseases caused by bacterial, parasitic and viral pathogens [2]. In particular, infection with *cyprinid herpesvirus 3*, also known as *koi herpesvirus*, induces koi herpesvirus disease (KHVD) which may be associated with high mortality rates and severe economic losses [5], and poses a potential risk for aquaculture of carp to maintain global food security. A further potential threat to carp aquaculture is “koi sleepy disease” (KSD) which is associated with an infection with *carp edema virus* (CEV). The disease is accompanied with lethargic behaviour (hence the name), congested gills [6], enophthalmos and skin alterations often around the mouth and at the base of the fins [7, 8]. Initially, disease outbreaks associated with significant losses (up to 99%) were observed in koi populations only, but infections and high mortality have also been recorded in farmed common carp in all age groups [6, 8–10]. KSD outbreaks were initially observed in the 1970’s in koi populations in Japan, and for a long time these appeared to be limited to this country [6, 7, 11]. However, in 1996 this disease was detected in the USA in koi [10]. Currently, CEV infections and KSD are reported from over at least 3 continents (Asia, North America and Europe). In Europe CEV was first detected in the UK by Way and Stone [8] from a disease outbreak in koi and common carp which had occurred in 2009 and 2012 respectively. This was followed by reports in koi from The Netherlands [12], and koi and cultured common carp from Austria [13], Germany [9, 14] and Poland [15], which suggests a wide distribution of this virus.

Not much is known about the virus that causes KSD. Besides several electron microscope images documenting poxvirus-like particles [6, 10, 14], only a fragment of the DNA sequence encoding the core protein P4a is published [15, 16]. Whole genome sequencing studies are underway [17] but currently a sequence has not yet been published. Initial data from phylogenomic analyses seem to confirm that CEV belongs to the *Chordopoxvirinae* subfamily of *Poxviridae* [17]. The known data for the P4a nucleotide sequence show over 6% variation between various isolates [8, 15]. This suggests the existence of at least two or even three genogroups or lineages of the virus [15]. The genogroup I was mainly associated with infections in farmed common carp, genogroup IIa was predominantly, but not exclusively, detected in koi [8, 15] while genogroup IIb was lately discovered in few carp samples from Poland [15]. CEV from genogroup IIa is presumed to have been distributed globally via the koi trade and probably has repeatedly been imported into Europe from Japan. This could have been non-detected because asymptomatic, CEV infected koi, may have entered Europe, as Japanese koi breeders are encouraged to treat and prevent for outbreaks of clinical KSD by increasing the water temperature and performing long lasting salt baths at a concentration of 0.5% [6] which does not guarantee eradication of CEV. The original sources of the CEV from the genogroup I and genogroup IIb still remains unclear.

Current reports on KSD outbreaks or on CEV replication rely on observations of spontaneous outbreaks of the disease [6, 10]. However, this approach gives only limited information on the development of the disease, the susceptibility of different fish species to infection and disease development, or on target tissues of the virus. This information is important in order to develop a containment strategy including a proper sampling protocol of tissues for diagnostic purposes. Therefore, in this study we conducted experimentally induced infections of naïve carp and koi with CEV, derived from diseased farmed carp and from diseased koi, suffering from KSD, respectively. We analysed the onset and progression of the disease and tested the main tissues for virus replication in common carp and koi.

Domestication of common carp has led to the establishment of numerous breeds/strains which offer a valuable variety of genetic resources [2]. Strains with a different genetic background may present a large difference in susceptibility to viral pathogens. This was evident in case of *cyprinid herpesvirus 3* infections causing KHVD [18–20]. Carp strains of Asian origin, in particular the Amur wild carp (AS) from the Amur River basin and the Ropsha scaly carp (Rop, a breed developed from AS crossed with European strains), were proven to be far less susceptible to KHVD than carp strains from European origin such as the Prerov scaly carp (PS) from the Czech Republic [18]. Exploring differences in the susceptibility of various carp strains to CEV infection and development of clinical KSD could also lead to a limitation of losses caused by CEV in carp aquaculture. We therefore included AS, Rop and PS strains into challenge experiments for an investigation into different susceptibilities of carp to an artificially induced CEV infection. Moreover, koi, an ornamental variety of carp, were included into the challenge experiments as koi, are extremely susceptible to KHVD [18] and one of the CEV genogroups could be associated more closely with koi.

Differences in the susceptibility of carp strains to virus induced diseases could be associated with differences in the induction of immune responses, in particular the innate immunity, such as a type I interferon (IFN) response which often plays a role in limiting infections with poxviruses during initial infection stages [21]. Interestingly, poxviruses are also known to employ an array of mechanisms to evade these responses [22]. In our study, we therefore additionally analysed the induction of interferon responses in various carp strains after artificially induced CEV infection.

The main challenge faced when studying CEV infections is the lack of a cell line which is suitable for virus replication in vitro. In the present study, we used cohabitation experiments of naïve carp with KSD affected farmed common carp and koi as an infection source for CEV. We also used primary cultures from fins and gills of naïve carp and koi for inoculation of tissue homogenates from CEV infected carp to test if these tissue cultures are suitable for in vitro replication of CEV.

In order to fill the gaps in the current knowledge with regard to the CEV infection process in common carp, the present study had the following objectives: (1) to define the target organ/tissue for CEV replication (2) to give insight into the possible use of this tissue for virus replication in vitro, (3) to evaluate a possible persistence of the virus in infected fish for extended periods of time, (4) to determine the differences in susceptibility of various common carp strains to CEV infection and follow the development of KSD during infections with two different genogroups (I and IIa) of CEV respectively, (5) to assess the association between resistance of carp to CEV and the magnitude type I IFN responses.

## Materials and methods

### Naturally infected donor fish

#### KSD affected farmed common carp

Live common carp (*n* = 17, mean weight of 903 ± 121 g) showing clinical signs characteristic of KSD (lethargic, “sleepy” behaviour, swollen gills, enophthalmos) were collected from the stock of a German carp breeder. This stock was suffering mass mortality and was confirmed several days earlier as infected with CEV from the genogroup I by means of an end-point PCR developed by the Centre for Environment, Fisheries and Aquaculture Science, CEFAS, UK [15] and subsequent sequencing of the PCR product (see Additional file 1 for sequence alignment and Additional file 2 for phylogeny). The carp were transported to the Fish Disease Research Unit (FDRU) at the University of Veterinary Medicine in Hanover, Germany. Upon arrival in the laboratory, 4 carp were euthanised by immersion into a 0.5 g L^−1^ tricaine (Sigma) solution, dissected and samples from gills, skin, liver, gut, heart, kidney, head kidney, spleen and brain were collected separately in RNA*later* for DNA and RNA isolation. Gill samples were fixed in 4% buffered formaldehyde for histology. The remaining carp (*n* = 13) were kept in a 400 L tank supplied with a constant flow of 100 L h^−1^ of tap water for use in subsequent cohabitation experiments. The constant flow through of tap water allowed performing the experiments at a water temperature of 10–12 °C which matched to the water temperature in the KSD affected carp pond.

#### KSD affected koi

Live koi (*n* = 3, mean weight of 102 ± 13 g) with clinical signs characteristic of KSD (lethargic behaviour, swollen gills) were collected from a private pond population experiencing severe losses and confirmed several days earlier as infected with CEV from the genogroup IIa by the previously mentioned end-point PCR and sequencing of the PCR product (see Additional files 1 and 2). The koi were kept in a 400 L tank supplied with a constant flow of 90 L h^−1^ of tap water for subsequent use in a cohabitation experiment at 11–13 °C, which matched to the water temperature of the pond from which the KSD affected koi came from.

### Naïve recipient common carp and koi

Naïve common carp strains Amur wild carp (AS), Ropsha carp (Rop), Prerov scaly carp (PS), and koi were obtained as feeding yolk sac fry from the University of South Bohemia in Ceske Budejovice, Faculty of Fisheries and Protection of Waters, located in Vodnany, Czech Republic. Each experimental stock was established by artificial reproduction of the appropriate carp strain by means of the protocol established by Kocour et al. [23] using a full-factorial mating scheme of three females with three males. The stocks were kept in a closed recirculation system supplied with tap water from their egg stage. Fry were transported to the FDRU and raised in a recirculation system filled with tap water at 20 °C and fed a commercial carp feed (Skretting, Norway) at 1% of body weight per day. At the start of the infection experiments the fish had a mean weight of 3.7 ± 0.9 g. Fish larvae from the same crossing were transported to the Experimental Fish Farm of The Stanislaw Sakowicz Inland Fisheries Institute in Olsztyn, located in Zator, Poland. There, the fish were kept in a recirculation system of the hatchery at 23 °C, which allowed a faster growth. The carp were transported to FDRU at a mean weight of 21.2 ± 2.2 g and placed at 20 °C in a flow through system 4 weeks prior to the infection experiment. All fish were raised and kept under virus and parasite free conditions. Prior to their use in infection experiments, all carp populations were confirmed to be free of DNA/RNA specific for CyHV-3, spring viremia of carp virus (SVCV), CEV and an as yet unclassified RNA virus with characteristics of Arena-, Ortho- and Paramyxovirus, which was recently detected by Granzow et al. [27] in carp suffering from gill necrosis. The carp were examined for the presence of these viruses by means of qPCR or RT-qPCR [15, 24–27]. The fish were also inspected for the presence of ectoparasites by means of fresh smears from skin and gill surfaces which were examined with a light microscope. Before cohabitation with donor fish having a natural CEV infection, all recipient fish were acclimatized to the water temperature in the infection tanks by lowering the water temperature from 20–12 °C by 1 °C per day.

### Cohabitation experiments

Cohabitation experiments I, II, III (Co I, II, III) were performed with CEV from the genogroup I followed by one cohabitation experiment IV (Co IV) with CEV from the genogroup IIa. All animal experiments were done according to national and international regulations for experimentation with animals and under approval of the Lower Saxony State Office for Consumer Protection and Food Safety under the reference number: 33.19-425 2-04-16/2144.

#### Infections with CEV from the genogroup I

In the first cohabitation experiment (Co I), 25 individuals each from the koi and the PS strain (body weight between 18 and 22 g) were cohabitated at 10–12 °C with the KSD affected common carp in a 400 L tank supplied with a constant flow (100 L h^−1^) of tap water. At 2, 4, 6, 10 and 15 days post-exposure, five fish per strain and per time point were euthanised by immersion into a 0.5 g L^−1^ tricaine (Sigma) solution. Pieces of gill, skin, kidney and head kidney were collected and stored in RNA*later* solution. Additionally, the same organs from two koi and two carp were sampled and put into 4% buffered formalin 6 days post-exposure.

In the second cohabitation experiment (Co II), carp from the strains AS, koi, PS, and Rop were used (mean body weights were between 2.7 and 4.1 g). From all four carp strains, 11 individuals were exposed to the KSD affected common carp in a 400 L tank supplied with a constant flow (100 L h^−1^) of tap water. At 6 and 11 days post-exposure, four individuals per strain were euthanised like described above. From all fish, gills were collected individually in RNA*later* for DNA and RNA isolation. As control for gene expression, gills collected from non-infected four fish per carp strain were used. Furthermore, at 11 days post-exposure, three fish per carp strain were euthanised as previously described and fixed with 4% buffered formaldehyde for histology.

In the third experiment (Co III), ten koi were exposed to carp (*n* = 3) which survived a CEV infection associated with clinical KSD. The koi were cohabitated to the carp three months post onset of the disease and one month after the last observation of clinical signs. After 9 days of cohabitation, all donor and recipient fish were euthanised and the gills were separately collected in RNA*later* for DNA and RNA isolations.

#### Infection with CEV from the genogroup IIa

In the fourth cohabitation (Co IV) experiment, carp from the strains AS, koi, PS, and Rop (mean body weight of 3.7 ± 0.9 g) were cohabitated with koi infected with CEV from the genogroup IIa. From all four carp strains, eight fish were cohabitated with KSD affected koi and at 6 and 11 days post-exposure, four individuals per strain were euthanised and their gills were aseptically collected in RNA*later*. Furthermore, at 11 days post-exposure, gills from three fish per strain were fixed with 4% buffered formaldehyde for histology. As negative control for gene expression and histology, gills collected from four non-infected fish per carp strain were simultaneously euthanised and sampled.

#### Testing for intercurrent infections

In the experiments Co II and Co IV, gills taken from four koi 11 days post-exposure were tested for presence of DNA/RNA specific of CyHV-3, SVCV and the unclassified RNA virus with characteristics of Arena-, Ortho- and Paramyxovirus as described above. Furthermore in the same experiments at 6 and 11 days post-exposure gills from randomly selected fish of all four strains were tested for the presence of ectoparasites.

### Preparation of skin and gill explants for primary cell cultures and subsequent infection with CEV

From naïve carp, which were sampled during health checks, fins and gills were collected into PBS supplemented with 100 IU mL^−1^ penicillin, 100 mg mL^−1^ streptomycin, 100 mg mL^−1^ gentamycin and 1 mg mL^−1^ amphotericin B (Sigma), and placed on ice. Fins were cut into small pieces (<10 mm^2^) and placed individually into the wells of 24 well tissue culture plates. 1 mL of culture medium (medium 199 supplemented with 20% FCS, 100 IU mL^−1^ penicillin, 100 mg mL^−1^ streptomycin, 100 mg mL^−1^ gentamycin and 1 mg mL^−1^ amphotericin B [Sigma]) was added to each well. Primary fin cultures were incubated at 25 °C in a humidified atmosphere containing 2% CO_2_. After 96 h, cultures reaching >50% confluence were selected for infection with CEV. Gill arches collected from the fish were cut into four pieces and each of the pieces was placed into a well of a 24 well tissue culture plate containing 1 mL of culture medium. These cultures were immediately used for infection with CEV. Cultures were prepared from three individuals of each of the carp strains AS, koi, Rop and PS.

CEV suspension for in vitro culture experiments was prepared by liberating the virus from the gills of common carp showing clinical signs of KSD according to a standard protocol [28]. Briefly: tissue was mechanically lysed in medium described above creating 10% w/v suspension and incubated over night at 4 °C. The tissue debris was removed by centrifugation at 1000 × *g* for 30 min at 4 °C. The supernatant was filter sterilised (0.45 μm pore size) and used for in vitro infections.

From each fish, two primary fin cultures and two primary gill explant cultures were incubated with the CEV suspension, and two wells with cultures from each organ received medium without virus as negative controls. The cultures were incubated for 48 h at 25 °C in a humidified incubator containing 2% CO_2_. Additionally, two gill explants per fish were infected and incubated for 48 h at 15 °C without CO_2_. After 48 h, the medium was removed from all cultures and the cells lysed in 1 mL Tri-Reagent (Sigma) before being transferred into 1.5 mL reaction tubes and stored at −80 °C until RNA isolation.

### DNA isolation

DNA was isolated from 25 mg of tissue, after mechanical lysis in a QIAgen Tissuelyser II (Qiagen), using the QIAamp DNA Mini Kit (Qiagen) according to the manufacturer’s instructions. After isolation, the samples were diluted to 50 ng μL^−1^ and stored at −80 °C.

### RNA isolation and cDNA synthesis

Total RNA was extracted using Tri-reagent (Sigma) in accordance with the manufacturer’s instructions. Any remaining genomic DNA was digested with 2 U of DNase I (Thermo Fisher Scientific) according to the manufacturer’s protocol. Synthesis of cDNA was performed from 300 ng of total RNA using the Maxima™ First Strand cDNA Synthesis Kit (Thermo Fisher Scientific). A non-reverse transcriptase control was included in the analysis of each sample. cDNA samples were diluted 1:20 with nuclease-free water prior to RT-qPCR analysis.

### qPCR/RT-qPCR

For detection and quantification of CEV from the genogroup I DNA, mRNA, and the analysis of gene expression, a SYBRGreen based qPCR/RT-qPCR was used. Reactions were performed in duplicate using the Maxima SYBR Green 2× mastermix (Thermo Fisher Scientific) in a Stratagene Mx3005P cycler (Agilent). The reaction mix was prepared as follows: 1× Maxima SYBR Green mastermix (with 10 nM of ROX), 0.2 μM of each primer (sequences in Additional file 3), 5.0 μL of DNA (50 ng μL^−1^) or 20× diluted cDNA and nuclease-free water to a final volume of 25 μL. The amplification program included an initial denaturation at 95 °C for 10 min, followed by 40 cycles of denaturation at 95 °C for 30 s, annealing at 55 °C for 30 s and elongation at 72 °C for 30 s. A dissociation curve was performed at the end of each run. For detection and quantification of CEV from the genogroup IIa DNA and mRNA, a qPCR based on a double labelled probe was used as described by Adamek et al. [26]. A recombinant DNA plasmids standard curve from 10^1^ to 10^7^ gene copies was prepared and used for quantification of the copy number from each sample as described by Adamek et al. [29].

For normalization of expression, the gene encoding the 40S ribosomal protein S11 was used as reference gene. The level of gene expression is shown as the copy number of the gene normalised against 1 × 10^5^ copies of the 40S ribosomal protein S11 (normalised copy number) according to the following formula:

Normalised copy number = mRNA copies per PCR for target gene/(mRNA copies per PCR for reference gene/10^5^).

### Histology

Euthanised fish were fixed in 4% buffered formaldehyde (Roth, Germany) and stored for 48 h at 4 °C. The entire fish or gill samples were embedded into paraffin wax according to a standard laboratory protocol. Sections were cut to a thickness of 3 µm and stained with haematoxylin and eosin (HE). For evaluation of morphological changes a semi-quantitative scoring system was applied. A high power field (hpf; magnification 400×) was used to quantify the number of mitotic figures and apoptotic cells, the results are presented as a number of affected cells per hpf. As control, gill samples collected from non-infected fish (not cohabitated with infected fish) of the different strains were used.

### Statistical analysis

SigmaPlot 12 software (Systat Software) was used for statistical analysis. Normalised gene expression data and virus load were transformed using a Log10(×) transformation before further statistical analysis. Significant differences (*p* ≤ 0.05) in virus load and gene expression during CEV infection were assessed using a 1-way or 2-way ANOVA with subsequent pairwise multiple comparisons using the Holm-Sidak method. Data are presented as box plots of 25–75% (±minimum and maximum values) with an indication of mean and median using Statistica 13 software (Dell Software).

## Results

### Virus load and replication in KSD affected carp

**Figure 1:**
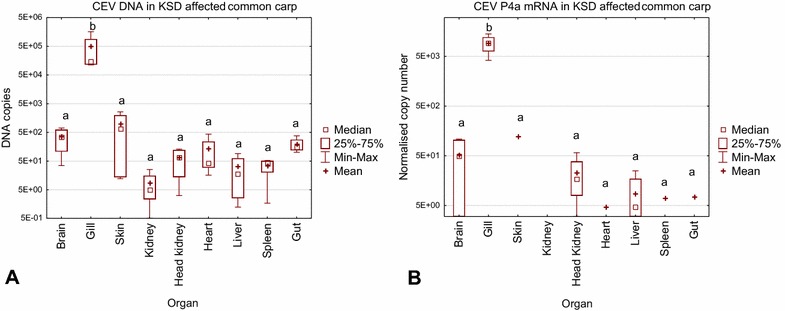
**CEV load and replication in tissues of common carp affected by KSD.** *Carp edema virus* load (**A**) was measured by qPCR as copy numbers of virus specific DNA. As a surrogate for CEV replication, the expression of the gene encoding the CEV core protein P4a (CEV P4a) was determined (**B**). The data on virus load and expression of viral mRNA are shown as box plots indicating the range of 25–75% in the box (±minimum and maximum values) of genome copies in 250 ng of isolated DNA from *n* = 4 fish (virus load), or as the copy number of mRNA encoding the viral P4a gene normalised against 100 000 copies of the carp 40S ribosomal protein S11. Symbols “+” and “□” indicate mean and median, respectively. Different letters indicate significant differences at *p* ≤ 0.05 between the carp tissues.

A quantification of CEV genome copy numbers in selected organs of KSD affected common carp revealed that in all individuals, gills harboured the highest number of CEV specific DNA copies with a mean of 504 000 copies and median of 147 000 copies per 250 ng of extracted DNA. The virus load in this organ was statistically significantly higher than the virus load in all other tissues (Figure 1).

All other organs had a virus load below 1000 copies per 250 ng of isolated DNA, with the skin having the highest (mean copy number 988; median 666 copies) whereas the kidney, head kidney, spleen and liver harboured less than 67 copies per 250 ng of isolated DNA.

**Figure 2:**
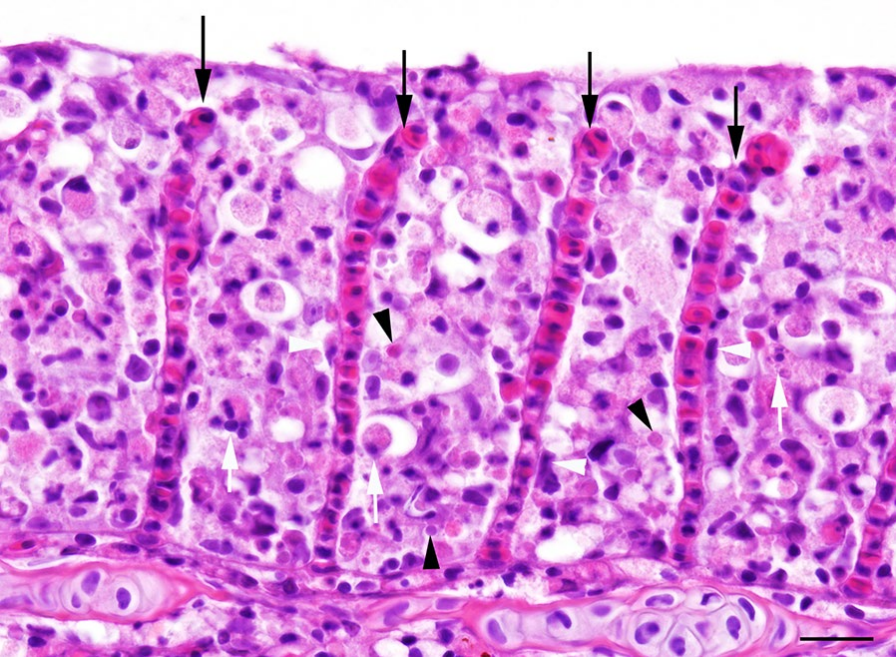
**Gills of KSD affected common carp (donor fish).** Clubbing and fusion of secondary gill lamellae with complete occlusion of the interlamellar spaces due to accumulation of cellular debris (white arrows), and hypertrophy of epithelial cells (white arrowheads); Note cytoplasmatic eosinophilic inclusions (black arrowheads); black arrows = lamellar capillaries; HE, bar = 40 µm.

Replication of the CEV virus was measured by relating the mRNA expression of the gene encoding for the CEV core protein P4a to a housekeeping gene of carp (40S ribosomal protein S11). The results are similar to those obtained for the virus load with a significantly higher virus replication in the gills (mean 924 and a median of 936 normalised copies). In samples from the heart, skin, spleen, and gut, virus replication could only be detected in one out of four carp tested with low copy numbers (less than 55 normalised copies) (Figure 1). Histological examination of the gills from five donor fish revealed similar severe changes characterized by clubbing and fusion of secondary gill lamellae. Interlamellar spaces were occluded due to hypertrophy and proliferation of epithelial cells (hyperplasia), accumulation of cellular debris and mild to moderate infiltration of eosinophilic granular cells (Figure 2). The number of mitotic figures varied between 1–2 and 3–5 high power field^−1^ (hpf; magnification 400×) in individual fish. Cell shrinkage, nuclear fragmentation, and chromatin condensation were indicative of apoptotic cell death. The number of apoptotic cells was > 15 hpf^−1^. Disseminated eosinophilic cytoplasmic inclusions, occasionally surrounded by a clear halo, were observed in epithelial cells which suggest viral inclusion bodies.

### Virus load and replication in recipient koi and common carp after cohabitation with carp infected with CEV from the genogroup I

In cohabitation experiment I (Co I), koi and PS carp were used. After cohabitation with KSD affected carp, single PS carp as well as koi showed lethargic behaviour at days 6 and 10. At day 15, most of the fish showed a behaviour which could be associated with KSD (Table 1). When virus load and virus replication were analysed in gills, skin, kidney and head kidney, the gills harboured the highest amount of virus specific DNA at all sampling dates. At 2 days post-exposure, the gills of koi had a mean copy number of 17 179, with a median 19 240, and the gills of PS carp had a mean of 3410 copies and median of 3389 copies (Table 2). After 4 days of infection, the virus load was significantly higher in koi compared to carp with a mean copy number of 68 441 copies in koi and a more scattered dispersal of the virus load, versus a mean of 395 copies in PS carp. At later time points, the virus loads were highly variable between individuals from both carp strains. Some individuals from both strains had extremely high values of virus loads, similar to those recorded in the KSD affected donor carp. At day 15 post-infection, most of the exposed fish showed a behaviour characteristic for KSD, and had high mean and median values for virus load, with 150 260 copies (mean) and 152 900 copies (median) in koi and 721 436 copies (mean) and 230 400 copies (median) in PS carp. These were similar to the levels which were observed in KSD affected donor carp. The same observations were made when the level of viral mRNA in gills was measured. At day 15 post-infection, a mean mRNA copy number of 2030 and a median of 2307 was seen in gill samples from koi and a mean copy number of viral mRNA of 16 343 (median 14 333) in gill samples from PS carp.

**Table 1:** Occurrence of KSD clinical sings during cohabitation experiments

Cohabitation experiment no.	CEV genogroup	Observation day	Development of KSD clinical signs (no. of fish)				Severity of histological changes			
			Koi	PS	Rop	AS	Koi	PS	Rop	AS
Co I	Genogroup I	15	5/5	4/5	n.d.	n.d.	+/++*	+/++*	n.d.	n.d.
Co II	Genogroup I	11	7/7	7/7	2/7	0/7	+/++	++/+++	++	+/++
Co IV	Genogroup IIa	11	4/4	0/4	0/4	0/4	+/++	+/++	+/++	++

Number of fish which developed clinical signs (lethargy, laying on the bottom of the tank) associated with KSD at the last day of cohabitation experiment, symbol “n.d.” indicates that this particular carp strain was not evaluated. Severity of histological changes which was semiquantitatively graded, which is indicated with following symbols: “−” no histological lesion; “+” mild histological changes; “++” moderate histological changes; “+++” severe histological changes. Symbol “*” indicates that histological analyses was performed at day 6, which was not the last day of infection.

**Table 2:** CEV load and replication in tissues from carp after experimental cohabitation with KSD affected common carp (Experiment no. Co I)

	Koi					PS				
	Day 2	Day 4	Day 6	Day 10	Day 15	Day 2	Day 4	Day 6	Day 10	Day 15
A (DNA)										
Gill										
Mean	1.72E+04	6.84E+04	2.10E+05	3.35E+05	1.50E+05	3.41E+03	3.95E+02	2.88E+05	3.71E+05	7.21E+05
Median	1.92E+04	3.19E+04	1.06E+04	2.28E+04	1.53E+05	3.39E+03	1.00E+02	1.10E+03	9.03E+03	2.30E+05
SD	1.10E+04	9.71E+04	3.15E+05	7.13E+05	1.34E+05	2.25E+03	6.26E+02	6.42E+05	6.85E+05	1.21E+06
Skin										
Mean	1.01E+02	4.90E+01*	3.72E+02	1.01E+02	1.13E+04	1.84E+01	9.20E+00*	2.72E+00	7.52E+01	1.44E+02
Median	2.90E+01	2.39E+01*	1.22E+02	8.61E+00	1.42E+03	6.13E+00	1.00E+00*	1.03E+00	7.13E+01	6.78E+01
SD	1.65E+02	7.67E+01*	5.42E+02	2.08E+02	2.21E+04	2.70E+01	1.83E+01*	4.42E+00	5.65E+01	1.69E+02
Head_Kidney										
Mean	4.81E+01	3.54E+01	1.99E+01	5.10E+02	2.94E+04	3.93E+00	9.06E+00	1.34E+01	1.52E+02	1.67E+02
Median	2.65E+00	1.14E+01	4.66E+00	2.05E+01	4.78E+02	0.00E+00	0.00E+00	2.61E+00	1.00E+00	0.00E+00
SD	7.38E+01	5.60E+01	3.78E+01	7.99E+02	6.44E+04	6.21E+00	2.03E+01	2.62E+01	3.16E+02	3.17E+02
Kidney										
Mean	0.00E+00	2.02E+01	8.30E+00	1.72E+00	1.38E+02	0.00E+00	0.00E+00	0.00E+00	5.35E+01	1.32E+02
Median	0.00E+00	0.00E+00	2.98E+00	0.00E+00	7.82E+00	0.00E+00	0.00E+00	0.00E+00	2.16E+01	1.06E+01
SD	0.00E+00	4.27E+01	1.51E+01	3.84E+00	2.86E+02	0.00E+00	0.00E+00	0.00E+00	7.90E+01	2.79E+02
B (RNA)										
Gill										
Mean	2.46E+02	4.15E+03	8.20E+03	8.74E+03	2.03E+03	1.01E+02	1.64E+01	3.11E+03	5.60E+03	1.63E+04
Median	1.52E+02	5.01E+02	6.96E+01	7.45E+02	2.31E+03	6.48E+01	7.84E+00	2.89E+00	5.20E+02	1.43E+04
SD	2.12E+02	7.65E+03	1.19E+04	1.84E+04	1.23E+03	1.00E+02	2.35E+01	6.94E+03	1.14E+04	1.91E+04
Skin										
Mean	0.00E+00	0.00E+00	1.89E+00	8.29E−01	0.00E+00	0.00E+00	2.27E−01	0.00E+00	0.00E+00	1.06E+00
Median	0.00E+00	0.00E+00	0.00E+00	0.00E+00	0.00E+00	0.00E+00	0.00E+00	0.00E+00	0.00E+00	0.00E+00
SD	0.00E+00	0.00E+00	2.84E+00	1.85E+00	0.00E+00	0.00E+00	5.07E−01	0.00E+00	0.00E+00	2.36E+00
Head Kidney										
Mean	2.00E−01	2.47E−01	2.00E−01	2.90E−01	2.20E+03	8.43E−01	0.00E+00	0.00E+00	9.69E−01	1.03E+00
Median	0.00E+00	0.00E+00	0.00E+00	0.00E+00	6.94E+00	0.00E+00	0.00E+00	0.00E+00	0.00E+00	0.00E+00
SD	4.47E−01	5.51E−01	4.47E−01	6.48E−01	4.90E+03	1.40E+00	0.00E+00	0.00E+00	1.66E+00	1.80E+00
Kidney										
Mean	4.92E+00	0.00E+00	2.09E+00	2.00E−01	1.36E+02	0.00E+00	0.00E+00	2.00E−01	0.00E+00	1.82E+02
Median	1.16E+00	0.00E+00	0.00E+00	0.00E+00	3.67E+01	0.00E+00	0.00E+00	0.00E+00	0.00E+00	2.01E+00
SD	7.50E+00	0.00E+00	4.15E+00	4.47E−01	2.61E+02	0.00E+00	0.00E+00	4.47E−01	0.00E+00	3.80E+02

*Carp edema virus* load was measured by qPCR as copy numbers of virus specific DNA and, as a surrogate for virus replication, the expression of the mRNA encoding the gene of the CEV core protein P4a (CEV P4a) was analysed in the gills, skin, kidney, and head kidney of koi and Prerov (PS) common carp during cohabitation with carp affected by KSD. Samples were collected 2, 4, 6, 10, 15 days post-exposure from *n* = 5 fish per day. The data on virus load (A) and expression of viral mRNA (B) are shown as mean, median and standard deviation (SD) of genome copies in 250 ng of isolated DNA from *n* = 4 fish (CEV load) or of the copy numbers of mRNA encoding the P4a gene normalised against 100 000 copies of the carp 40S ribosomal protein S11 (CEV replication). Symbol “*”indicate significant differences at *p* ≤ 0.05 between carp strains.

Histological analyses of gills at day 6 post-exposure showed similar, however less severe, morphological changes than seen in the KSD affected donor fish. A partial oedema of respiratory epithelia could be observed which resulted in an incomplete fusion of secondary gill lamellae caused by a hyperplasia of interlamellar cells.

Other tissues were only marginally positive for CEV DNA and for viral mRNA. In samples from day 15, however, some experimentally infected individuals had a higher virus load in the kidney than was seen in the KSD affected donor carp (Table 2).

### Susceptibility of carp strains to CEV infection and development of KSD (CEV genogroup I)

The cohabitation (Co II) of naïve common carp from the strains AS, Rop, PS and koi with common carp infected with CEV (genogroup I) showed significant differences in susceptibility to the infection, with koi and PS being more susceptible than Amur wild carp. Among the koi and PS carp, some individuals displayed clinical KSD with lethargic behaviour and an over-secretion of skin mucus 5 days post onset of cohabitation, and at day 7 some koi were laying at the bottom of the tank for a couple of seconds. From day 10 onwards, most of the koi and PS carp, and a few Rop carp, were resting on one side of their body at the bottom of the tank (Table 1). At the same time, fish from the AS strain did not show any noticeable behavioural clinical signs of infection besides indicators of stress (increased activeness and mucus production). Histology of PS and Rop carp showed marked differences between infected and non-infected fish (Table 1). The gills of infected fish displayed a moderate to severe increase of interlamellar cells, most likely epithelial cells, up to the tips of the secondary lamellae. In contrast, a mild to moderate proliferation of interlamellar cells was observed in AS and koi. The number of apoptotic cells was higher in Rop and PS carp (5–15 hpf^−1^) compared to AS and koi (<5 hpf^−1^). Similarly, the number of mitotic figures was higher in ROP and PS carp (3–5 hpf^−1^) compared to AS and koi (1–2 hpf^−1^). Rop and PS carp showed a moderate infiltration of the gills with eosinophilic granular cells, whereas in AS and koi a mild to moderate infiltration was recorded. Eosinophilic inclusions in epithelial cells were observed only in single cells of two out of three Rop carp.

**Figure 3:**
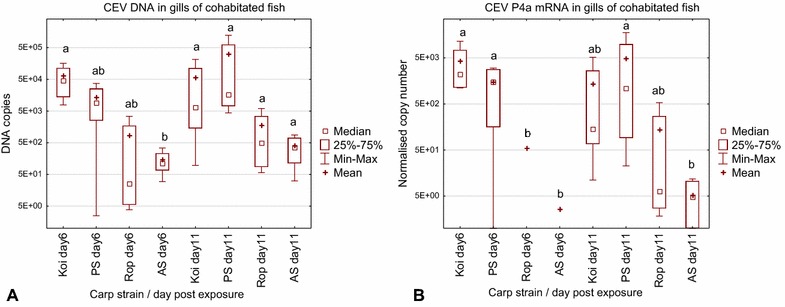
**Susceptibility of different strains of carp to an infection with CEV from the carp genogroup I (Experiment no. Co II).** Depicted is *carp edema virus* load (**A**) and replication (**B**) in the gills of koi (Koi) and common carp from the strains Amur wild carp (AS), Ropsha (Rop) and Prerov (PS) during cohabitation with carp affected by KSD. CEV load was measured by qPCR as copy numbers of virus specific DNA. As a surrogate for CEV replication, the expression of mRNA encoding the gene of the CEV core protein P4a (CEV P4a) was determined. The data on virus load and expression of viral mRNA are shown as box plots indicating the range of 25–75% of the values in the box (±minimum and maximum values) of genome copies in 250 ng of isolated DNA from *n* = 4 fish (virus load) or of the copy number of mRNA encoding the viral P4a gene normalised against 100 000 copies of the carp 40S ribosomal protein S11 (CEV replication). Symbols “+” and “□” indicate mean and median respectively. Different letters indicate significant differences at *p* ≤ 0.05 between carp strains.

The quantification of virus DNA and of viral mRNA confirmed the observed clinical signs (Figure 3A, B). After 6 days of cohabitation, the gills of koi harboured a mean of 64 125 copies of viral DNA per 250 ng of DNA with median of 44 440 copies. At the same time, the gills of Amur wild carp (AS) harboured only 150 copies with median of 114 copies, significantly less than in the koi samples. In the gills of PS carp, a mean virus load of 13 715 copies and a median of 8944 copies were measured, and in the gills of Ropsha carp a mean virus load of 866 copies with a median of 25 copies was seen. At 11 days post-exposure, the mean virus load in the gills of koi had decreased with a mean of 56 522 copies and a median of 6446 copies, while in the gills of PS carp the highest mean virus load was measured with 316 620 copies and a median of 16 055 copies. The virus loads in AS and Rop were noticeably, but not significantly, lower with a mean number of 1753 copies and a median of 492 copies in Rop and a mean of 408 copies and a median of 361 copies in AS carp.

The differences in virus susceptibility between the carp strains were higher when viral replication was measured (Figure 3B). At day 6 post-exposure, after normalisation koi had a mean mRNA copy number of 4278 (with a median of 2227) and PS carp had a mean of 1493 normalised copies of viral mRNA (with median of 1485). In Rop and AS viral mRNA was detected only in the gills of one out of four carp with 218 and 10 normalised copies, respectively. This was significantly lower when compared to koi and PS. After 11 days post-exposure, individuals from the PS strain had the highest virus mRNA level with 4945 normalised copies (median of 1078), koi had a mean level of 1360 normalised copies (median of 143) while Rop and AS had a lower virus mRNA level of 137 (median 6) and 5 (median 5) copies of viral mRNA (Figure 3B). The difference in the level of viral mRNA between PS and AS was statistically significant.

### Susceptibility of carp strains to CEV infection and development of KSD (CEV genogroup IIa)

The cohabitation (Co IV) of naïve koi and common carp from the strains AS, Rop, and PS with CEV infected koi (CEV genogroup IIa) revealed significantly different features compared to an infection of these strains with CEV from the genogroup I. Koi were the most susceptible to infection with CEV from the genogroup IIa and were the only fish to develop clinical signs of KSD. These fish started to show apathetic behaviour by day 4 post-exposure. At the same time, an over-secretion of skin mucus could be observed. By day 6, individual koi were laying at the bottom of the tank for a couple of seconds. From day 8 onwards, all koi were laying at the bottom of the tank for most of the time, and one koi was euthanised at day 10 because it stopped moving. Carp from other strains (AS, PS and Rop) developed no noticeable differences to their normal swimming behaviour (Table 1). In all common carp strains and in koi, similar mild to moderate lesions were found during histological analyses (Table 1). There was a proliferation of interlamellar cells with partial or total occlusion of the interlamellar space. A few cells showed hypertrophy and the number of apoptotic cells was low (<5 hpf^−1^). The number of mitotic figures was low (1–2 hpf^−1^) to none. All fish showed a mild infiltration of the gills with eosinophilic granular cells. Eosinophilic inclusions in epithelial cells were not observed.

**Figure 4:**
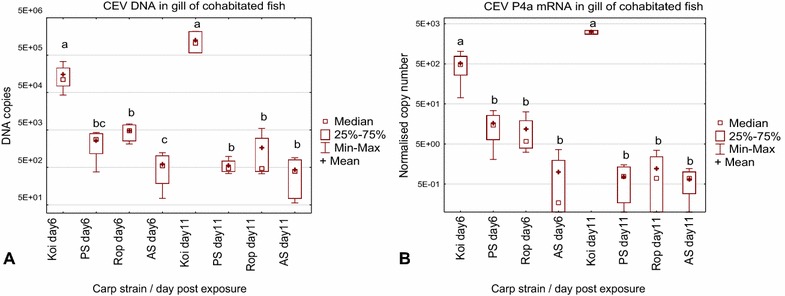
**Susceptibility of different strains of carp to an infection with CEV from the koi, genogroup IIa (Experiment no. Co IV).** Depicted is *carp edema virus* load (**A**) and replication (**B**) in the gills of koi (Koi) and common carp from the strains Amur wild carp (AS), Ropsha (Rop) and Prerov (PS) during cohabitation with koi affected by KSD. CEV load was measured by qPCR as copy numbers of virus specific DNA. As a surrogate for CEV replication, the expression of mRNA encoding the gene of the CEV core protein P4a (CEV P4a) was determined. The data on virus load and expression of viral mRNA are shown as box plots indicating the range of 25–75% of the values in the box (±minimum and maximum values) of genome copies in 250 ng of isolated DNA from *n* = 4 fish (virus load) or of the copy number of mRNA encoding the viral P4a gene normalised against 100 000 copies of the carp 40S ribosomal protein S11 (CEV replication). Symbols “+” and “□” indicate mean and median respectively. Different letters indicate significant differences at *p* ≤ 0.05 between carp strains.

The behavioural observations were confirmed as KSD by virus quantification (Figure 4A). After 6 days of cohabitation, gills of recipient koi harboured 151 668 copies of virus specific DNA (mean, median 112 600 copies) per 250 ng of DNA. This was a significantly higher virus load than in the gills of other carp strains. The gills of Amur wild carp (AS) had the lowest number of CEV specific DNA copies (mean: 604, median 555 copies), the PS carp had 2580 copies (mean, median 2826 copies) and Ropsha had 4728 copies (mean, median 4775). Interestingly, by day 11, the mean virus load had increased in the gills of koi to 1 253 267 copies (median 1 033 000 copies) while it decreased in the other carp strains to 436 copies (median 394 copies) in AS carp and 562 copies (median 461 copies) in PS carp. The virus load in Rop was the highest among the non-ornamental carp strains with a mean of 1691 copies (median 465 copies).

The difference in CEV susceptibility between koi and the other strains was even more evident when viral mRNA levels were measured as a surrogate for virus replication (Figure 4B). At day 6 post-exposure, koi had mean level of 514 normalised copies of viral mRNA (with median of 477), which increased to 3080 normalised copies (with median of 3062) by day 11 post-exposure. At both time points, this was significantly higher than the viral mRNA level in the gills of all other carp strains, which harboured mRNA levels from 16 to below 1 normalised copy (median 15 to below 1). Carp from the AS strain had less than 1 normalised copy of mRNA at both time points and proved to be least susceptible to an infection with CEV from the genogroup IIa.

### Intercurrent infections

In the experiments Co II and Co IV, gills of koi at 11 days post-exposure were confirmed to be free of DNA/RNA specific of CyHV-3, SVCV and the unclassified RNA virus with characteristics of Arena-, Ortho- and Paramyxovirus. This excludes a possible transfer of viruses other than CEV to the recipient fish during the cohabitation experiments. Furthermore gills of recipient fish were confirmed to be free of ectoparasites at 6 and 11 days post-exposure during both experiments, which excludes the development of a parasitic infection mimicking clinical signs of KSD.

### In vitro replication of CEV in explant cultures from gills and in primary cell cultures from fins

**Figure 5:**
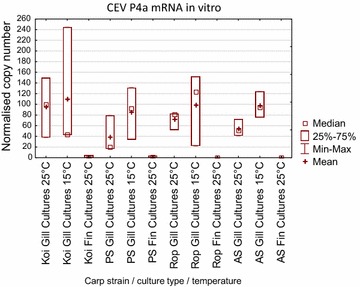
**Replication of CEV in gill explant cultures and in primary fin cultures of various carp strains.** Cultures were obtained from koi (Koi) and common carp from following strains: Amur wild carp (AS), Ropsha (Rop) Prerov (PS). Cultures were infected with CEV from the carp strain for 48 h at 15 or 25 °C. The CEV was re-isolated from KSD affected carp. As a surrogate for virus replication, the expression of mRNA encoding the viral core protein P4a (CEV P4a mRNA levels) is shown as mean (+SD) copy number of the P4a gene normalised against 100 000 copies of the carp 40S ribosomal protein S11 from *n* = 3 cultures.

In the explanted gill cultures virus replication, indicated by mRNA expression, could be detected after 48 h of infection in cultures from all carp strains with copy numbers between 38 and 110 normalised copies (Figure 5). There were no differences in mRNA levels between cultures from different carp strains. In contrast to this, CEV replication could not reliably be detected in primary fin cultures. Extremely low virus replication (2 normalised copies) could be recognized only in cultures derived from koi and PS-carp fins. No significant difference was seen in virus replication at different incubation temperatures (15 and 25 °C).

### Persistence of CEV in carp which survived KSD

A possible persistence of CEV in carp which survived clinical KSD was analysed by cohabiting 10 naïve koi with 3 donor carp (Co III). After 9 days of cohabitation, the donor carp and the recipient carp were euthanized and the gills of all carp were analysed for the presence of CEV DNA. In samples from naïve fish as well as in the donor carp, CEV DNA could not be detected.

### Type I IFN responses in relation to CEV infection and development of KSD

To test the hypothesis that differences in the susceptibility of carp strains to a CEV infection might be related to a different induction of type I IFN responses, the mRNA expression of the genes encoding interferon a2 and the interferon induced proteins viperin and RNA dependent protein kinase (PKR) were analysed in the gills of carp strains AS, koi, Rop and PS after infection with CEV from the genogroups I and IIa.

During the course of an infection with CEV from the genogroup I, a significant upregulation of the expression of the gene encoding IFN a2 was not observed in fish from any carp strain analysed (Table 3). In AS carp, a downregulation of IFN a2 expression was noticed at day 6 and 11 when compared to uninfected controls. The level of IFN a2 expression in uninfected control AS carp was highly variable and noticeably (but not statistically significant) elevated relative to the measurements of control carp from the other studied strains. The mRNA expression of the genes encoding for the interferon induced proteins viperin and PKR was strongly and statistically significantly upregulated in the gills of all carp strains at both time points when compared with uninfected controls. However, the expression level in the most resistant AS carp was significantly lower than in the more susceptible PS or koi (Table 3).

**Table 3:** mRNA expression of genes involved in the type I interferon response of common carp

Carp strain	CEV genogroup I			CEV genogroup IIa		
	Control	Day 6	Day 11	Control	Day 6	Day 11
Cyca IFN a2						
Koi						
Mean	*6.1E+01* a	*1.9E+02* a	*1.4E+02* a	*6.1E+01* a	*1.8E+02* a	*8.4E+02* b*
SD	3.3E+01	1.2E+02	9.5E+01	3.3E+01	1.0E+02	1.9E+02
PS						
Mean	*6.6E+01* a	*1.5E+02* a	*1.2E+02* a	*6.6E+01* a	*1.2E+02* a	*9.5E+01* a
SD	7.5E+00	2.4E+01	7.1E+01	7.5E+00	4.9E+01	3.3E+01
Rop						
Mean	*7.9E+01* a	*1.3E+02* ab	*9.6E+01* ab	*7.9E+01* a	*9.3E+01* a	*8.5E+01* a
SD	4.1E+01	4.3E+01	4.2E+01	4.1E+01	4.0E+01	4.4E+01
AS						
Mean	*1.6E+02* a	*4.8E+01* b*	*3.8E+01* b*	*1.6E+02* a	*1.2E+02* a	*1.2E+02* a
SD	1.2E+02	1.9E+01	1.8E+01	1.2E+02	4.7E+01	4.2E+01
Cyca Viperin						
Koi						
Mean	*7.3E+02* a	*4.2E+04* a*	*3.6E+04* a*	*7.3E+02* a	*3.1E+04* a*	*2.5E+04* a*
SD	3.2E+02	1.5E+04	2.1E+04	3.2E+02	1.0E+04	5.3E+03
PS						
Mean	*9.0E+02* a	*3.8E+04* a*	*2.7E+04* ab*	*9.0E+02* a	*9.4E+03* b*	*3.6E+03* b*
SD	2.6E+02	2.6E+03	1.3E+04	2.6E+02	4.4E+03	1.2E+03
Rop						
Mean	*9.0E+02* a	*2.3E+04* a*	*1.2E+04* ab*	*9.0E+02* a	*1.5E+04* ab*	*4.0E+03* b*
SD	6.7E+02	1.8E+04	1.0E+04	6.7E+02	1.9E+03	2.8E+03
AS						
Mean	*2.2E+03* a	*6.3E+03* b*	*7.7E+03* b*	*2.2E+03* a	*7.0E+03* b*	*1.4E+03* b*
SD	2.9E+03	5.2E+03	4.0E+03	2.9E+03	1.6E+03	2.4E+02
Cyca PKR						
Koi						
Mean	*3.6E+02* a	*5.6E+03* ab*	*4.5E+03* ab*	*3.6E+02* a	*5.4E+03* a*	*4.8E+03* a*
SD	8.4E+01	9.8E+02	2.2E+03	8.4E+01	1.6E+03	1.1E+03
PS						
Mean	*7.3E+02* a	*7.5E+03* a*	*4.9E+03* a*	*7.3E+02* a	*3.1E+03* ab*	*1.1E+03* bc*
SD	1.2E+02	7.6E+02	5.0E+02	1.2E+02	1.6E+03	2.1E+02
Rop						
Mean	*9.3E+02* a	*3.6E+03* bc*	*2.6E+03* ab*	*9.3E+02* a	*4.7E+03* a*	*1.7E+03* b*
SD	2.2E+02	1.3E+03	1.2E+03	2.2E+02	9.4E+02	1.0E+03
AS						
Mean	*7.4E+02* a	*2.0E+03* c*	*1.9E+03* b*	*7.4E+02* a	*2.0E+03* b*	*6.5E+02* c*
SD	5.8E+02	1.0E+03	7.2E+02	5.8E+02	5.0E+02	1.1E+02

Expression levels of genes encoding for common carp *Cyprinus carpio* (Cyca) virus-induced interferon a2 (Cyca IFN a2), the interferon induced proteins viperin (Cyca Viperin), and RNA dependent protein kinase (Cyca PKR) were measured in the gills during infection with *carp edema virus* from the genogroup I or genogroup IIa. Expression levels are shown as means and standard deviations (SD) of the copy number of mRNA encoding the gene normalised against 100 000 copies of the common carp 40S ribosomal protein S11 from *n* = 4 fish. Symbol “*” indicates significant differences at *p* ≤ 0.05 between infected and control individuals. Different letters indicate significant differences at *p* ≤ 0.05 between carp strains.

Infections with CEV from the genogroup IIa induced a statistically significant upregulation of IFN a2 mRNA expression in koi at 11 days post-infection but not in other carp strains when compared to uninfected controls (Table 3). Likewise, the mRNA expression of the gene encoding the interferon induced proteins viperin and PKR was mostly upregulated in koi at 6 and 11 days post-infection. The level of mRNA encoding for these proteins was also upregulated in carp from the other strains (especially at day 6 post-infection) however, the magnitude of upregulation was significantly lower in particular at day 11 post-infection (Table 3).

## Discussion

In our study, we showed that farmed common carp, as well as its ornamental variety koi, suffering from koi sleepy disease are able to transmit the viral disease to other fish of both varieties, causing similar clinical signs: laying on the bottom of the tank in a lethargic state, and the development of oedematous gills associated with a fusion of secondary gill lamellae. In addition, our study confirms, that during cohabitation an infective agent is transmitted, a putative poxvirus named *carp edema virus*. This is in line with the results of other colleagues, including previous attempts with cohabitation trials [17].

Based on molecular analysis of tissues from KSD affected individuals as well as from recipient fish during cohabitation with naturally infected fish, the gills were identified as the main target tissue of the virus. In gills, but not in other tissues from infected individuals, poxvirus-like particles were detected by electron microscopy [6, 10, 14]. Furthermore, the analysis of the virus load and the presence of viral mRNA by quantitative PCR in the current study underline that, whilst the gills are the main target, other tissues play a minor role as virus targets. The in vitro studies within this present investigation further support the view that the gills are the target tissue of the virus. Primary cultures from gills enabled CEV replication over a period of 2 days, but not in cultures from fins (present study) or cell lines derived from other tissues of carp, including fins and brain [14].

Our data provide evidence that CEV is one of the factors, responsible for the development of KSD. In addition to the detection of CEV in koi and carp suffering from KSD, the clinical signs of KSD and associated histopathological changes to the gills repeatedly developed in recipient fish which then harboured a high virus load in their gill tissue.

In previous studies, carp displaying pathological changes of the gills were found to be infected with several other viral pathogens, including a herpesvirus, a rhabdovirus and an ortho- and paramyxovirus [30]. In the current investigation, co-infections by other viral or parasitic pathogens could be excluded.

The morphological alterations in donor carp were characterised by a severe destruction of the gills, and were in line with the clinical disease of the animals. However, in the different fish strains of the cohabitation infections, the severity of the morphological changes correlated only partially with the development of the clinical disease. The relatively serious gill alterations observed in the Rop carp of experiment Co II would suggest the manifestation of a clinical disease but the behaviour of these fish remained largely unchanged. In contrast, lesions in koi of the same experiment were only mild to moderate, but the fish showed a sleepy behaviour. Likewise, this mismatch between clinical signs and severity of morphological gill lesions was also observed in koi from the cohabitation experiment Co IV. From the current data, factors responsible for the manifestation of clinical signs of KSD cannot be determined. The discrepancies between clinical signs and morphological alterations of gill tissues might be explained by metabolic disturbances that are not correlated to the morphological changes, however it could also suggest that pathological changes might not be the most reliable indicators of KSD. This needs to be investigated in further studies.

The destruction of the respiratory epithelium and the loss of interlamellar spaces in the gills might cause the fish suffering from hypoxia, however, those fish usually swim close to the water surface which was not observed during the present cohabitation experiments. Apart from respiration, gills play a role in the fish`s hydro-mineral balance and in its ammonia excretion process [31]. In carp infected with CyHV-3, which also affects the gills, the osmotic balance was disturbed [32, 33] and Atlantic salmon affected by amoebic gill disease (AGD) have been shown to suffer from acidosis [34]. In CEV infected koi and carp, patho-physiological studies are needed, which would include an analysis of the hydro-mineral balance, respiration and ammonia excretion, to explain the observed clinical signs.

In vertebrate hosts, even closely related poxviruses can display highly diverse host ranges and virulence [35]. In the present study, fish from several genetic strains of common carp were exposed to CEV from two genetically and biologically distinct genogroups I and IIa infecting mostly common carp or koi, respectively. Based on P4a sequence and epidemiological analyses, both of these viruses seem to be highly virulent and were associated with several cases of mass mortalities in carp or koi populations in Germany (data not shown). Our cohabitation experiments revealed remarkable differences in the susceptibility of carp of different genetic origin to infection with CEV and the development of clinical KSD symptoms (summarised in Table 1). In addition, these experiments revealed possible differences in the virulence between CEV genogroups which were associated with infections in koi compared to virus from the genogroup associated with infections in farmed carp in the field. While koi were clinically affected during infections with CEV from the genogroup IIa and were harbouring significantly higher virus loads and virus replication rates, this virus was cleared from the gill tissue of recipient fish from the other carp strains after an initial infection had occurred. In contrast to this, CEV from the genogroup I induced KSD in all koi and carp from the Prerov carp strain and very few carp from the Rop strain, while the disease did not manifest in any Amur wild carp. The infection, however, caused relatively serious morphological changes in the gills of fish from the Ropsha strain, while it did not affect the Amur wild carp population. Furthermore, koi and PS harboured significantly higher virus loads and replication than AS. Similar results were obtained in infections with the *ectromelia virus*, which induces mouse pox, when the infection process was analysed in susceptible and resistant strains of mice. Relative to mice from the susceptible strain, infection in liver of resistant mice (the main target organ) was suppressed from the third day post-infection onwards, showing lower virus replication [36].

Poxviruses replicate in the cytoplasm of host cells and, for the establishment of a permissive infection, require successful manipulation of the host’s antiviral immune system, in particular the innate immune response [37]. Therefore, we hypothesized that the drastic differences in CEV load and replication in carp with divergent genetic background could be related to differences in the magnitude of their innate immune responses. To test this hypothesis, analysis of the type I IFN response as a crucial factor for restricting virus replication by the induction of an antiviral state of cells was performed. In mice, the clearing of poxvirus (*vaccinia virus*) infections is associated with robust interferon responses [21, 38], and in fish this response also provides protection against VHSV, a OIE and EU notifiable rhabdovirus infecting rainbow trout [39]. However, in our CEV infected carp, the level of type I IFN responses seemed to be positively correlated with the virus load in infected fish. Therefore, similar to the CyHV-3 infection in carp [40], type I IFN cannot be associated with a higher resistance of carp strains to a CEV infection.

Recently, CEV has been related to significant losses in common carp aquaculture [9]. The higher resistance of fish from the AS strain for two genogroups of CEV, as reported in our study, provides a possibility to resolve this problem. Carp farms could implement programmes of i) single or more stage crossbreeding of less susceptible or CEV resistant carp strains with CEV susceptible carp strains, or ii) selective breeding of resistant carp strains and/or individuals, families etc. For example, experiments in mice strains have shown that the resistance to mouse pox is a dominant trait, which would favour selective breeding programmes [41, 42]. At present, such breeding programs for carp already exist using AS and Rop carp strains as a basis for the development of carp strains with a higher resistance to KHVD (CyHV-3 infection). The program based on the use of AS is conducted at the Faculty of Fisheries and Protection of Waters, University of South Bohemia in Ceske Budejovice, using the facilities in Vodnany, Czech Republic [43]. A similar program based on the AS and Rop strains and the local Zator strain has started in the Experimental Fish Farm of The Stanislaw Sakowicz Inland Fisheries Institute in Olsztyn, Poland. The fish from these breeding programmes could also be used to try to limit potential losses related to KSD. The latter program already aims at the limitation of losses in carp farming caused by both viral diseases KHVD and KSD.

Stocking of farm ponds with less susceptible carp strains or crosses may reduce the economic risk related to CEV infection, as our results indicate that the virus does not seem to persist in fish after its recovery from the disease. In our experiments, the presence of the virus could not be confirmed in fish surviving clinical KSD by qPCR one month after the last clinical signs had been recorded. These fish did not transfer the virus to naïve recipient fish. This might indicate that infected fish clear the virus and do not develop a persistent subclinical infection. However, this aspect of CEV biology needs to be investigated more thoroughly, in particular in experiments which explore the influence of stress on virus shedding of fish that have recovered from a clinical KSD. Furthermore, these studies should include screening of additional tissues where persistence could be more likely e.g. central nervous system tissues or gut-associated lymphoid tissue.

Analyses of virus load and virus replication in different organs of KSD affected fish, as well as in experimentally infected common carp and koi, underlined that, among the tissues investigated the gills are the main organ for CEV replication. The gill epithelium cells were reported to be the most affected in previous histopathological studies [6, 44]. Interestingly, the only two other members of poxviruses that attack fish, the *salmon gill poxvirus* (SGPV) and the putative poxvirus of ayu (*Plecoglossus altivelis*) also primarily infect gill epithelia [45, 46]. Hence, it could be speculated that these fish poxviruses might employ some unique mechanisms to infect and replicate in the gill epithelium. This could be supported by our in vitro experiments where only gill explant cultures were able to support CEV replication. In mammals, certain cell lineages or even differentiation states of cells were essential for the replication of particular poxviruses, for instance the *molluscum contagiosum virus* while others, like the *vaccinia virus* could replicate in a broad spectrum of cells (for review see: [47]). A very narrow tissue tropism also has a fundamental influence on the cell culture based virus diagnostic and in vitro replication. As most of the cell lines derived from common carp (e.g. common carp brain—CCB and koi fin 1—KF-1 cells) are fibroblasts and were established from other organs than gills [48, 49], the re-isolation of CEV from infected tissues in cell cultures will be challenging. In our hands, CCB cells failed to allow replication of CEV, despite several attempts [14].

This study aimed to give insights into the CEV infection process. We performed cohabitation experiments, in which CEV (from common carp and koi, respectively) was transferred from KSD affected fish to naïve common carp and koi. We estimated the virus load and viral mRNA expression in tissues of the recipient fish and linked these data with recorded histological changes. Our findings further showed that koi sleepy disease (KSD) in common carp and koi is caused by an infection with the *carp edema virus* (CEV). More importantly, the experiments show for the first time significant differences in the virulence between CEV genogroups, with higher virulence seen towards the same fish variety (common carp or koi) as the donor fish. Quantitative assessment of viral replication and virus load identified the gills as the target organ of CEV, which correlates with the occurrence of oedema in this organ after CEV infection. We explored the susceptibility of different carp strains to infection and found indications that Amur wild carp are relatively more resistant to infection with CEV and do not develop clinical signs for KSD, while in particular Prerov scaly carp and koi contained high virus loads and developed clinical symptoms of KSD. In contrast, Ropsha carp developed relatively serious morphological changes during infection with CEV from the genogroup I but were less susceptible to clinical KSD. We were not able to relate the resistance of certain carp strains to the infection to a higher type I IFN response of affected tissues. Despite not having a mechanistic explanation for the resistance to KSD, we do recommend using resistant strains of carp in breeding programs which could limit potential losses caused by this viral disease in aquaculture.

## Additional files

**Additional file 1.** **DNA sequence alignment presenting a nucleotide fragment encoding for the P4a core protein.** Sequences obtained from the CEV from the genogroup I and the CEV from the genogroup IIa used in the cohabitation experiments. Sequence alignment was performed with Clustal Omega.

**Additional file 2.** **Phylogenetic tree based on DNA sequences encoding for a 373 bp fragment of the P4a core protein of** ***carp edema virus***. Sequences published by Matras et al. [15] were obtained from GenBank. The positions of the sequences obtained from viruses used for the cohabitation experiments are indicated with arrows. Analysis was performed with PhyML 3.0 software based on the maximum-likelihood principle while tree rendering was performed with TreeDyn 198.3.

**Additional file 3.** **Sequences of primers used in this study.** Primers marked with “L” were used in qPCR for an estimation of virus load, primers marked with “E” were used in RT-qPCR expression analyses, primers marked with “P” were used for the amplification of gene fragments for plasmid based quantification. Primers marked with “S” were used for the amplification and sequencing of P4a gene fragment of CEV donor fish.
