# Supplementary material for: Experimental infections of different carp strains with the carp edema virus (CEV) give insights into the infection biology of the virus and indicate possible solutions to problems caused by koi sleepy disease (KSD) in carp aquaculture
Source: Vet Res. 2017 Feb 21;48:12. doi: 10.1186/s13567-017-0416-7 (PMC5320791; doi:10.1186/s13567-017-0416-7)
Supplement: Supplementary file 1 — Additional file 1. DNA sequence alignment presenting a nucleotide fragment encoding for the P4a core protein. Sequences obtained from the CEV from the genogroup I and the CEV from the genogroup IIa used in the cohabitation experiments. Sequence alignment was performed with Clustal Omega. [file 13567_2017_416_MOESM1_ESM.docx]

**CEV genogroup IIa TTGTTACCTTTTGTAGTTGTTTAATATTTGTGATAAGATTTCCATTAGCATAAAATCCTT**

**CEV genogroup I TTGTTACTTTTTGTAGTTGTTTAATATTTGTGATAAGATTTCCATTGGCATAAAATCCTT**

******* ************************************** *************

**CEV genogroup IIa CCCAAATTTGTGTTGATACATGTTTTAGTGTTTTGTAGATTGTAGCATTTCCTAGTTTGT**

**CEV genogroup I CCCAGATTTGGGTTGAAACATGTTTTAGAGTTTTGTATATTGTAGCATTTCCTAGTTTGT**

**** ***** ***** *********** ******** **********************

**CEV genogroup IIa ATGGCAAGAAACAAACTCTCTTTACTGCAACTCCTTGAGGAATTTGATCTAGAATTCCAC**

**CEV genogroup I ATGGCAAGAAACAAACTCTCTTTACTGTAACTCCTTGAGGAATCTGATCTAGAATTCCAC**

*************************** *************** ****************

**CEV genogroup IIa AGAATGTAATCTCAAATTTGTTTGTAGAGTTTTTGAAGTATACTGTTTCATCATACAATC**

**CEV genogroup I AATATGTAATCTCAAATTTGTTTGTGGAGTTTTTGAAATATACTACTTCATCATACAATC**

* ********************** *********** ****** **************

**CEV genogroup IIa CTAGAACTAGAGCAAGATTAGAAGTCATTGTCTTATCGAAGACATTCATCTTATTCCAAT**

**CEV genogroup I CTAGAACTAGAGCAAGATTAGAAGTCATTGTCTTGTCAAAGACAGACATCTTATTCCAAT**

********************************** ** ****** **************

**CEV genogroup IIa CATCAATCTGAATTCCTTTCCAGAACATAGCATTTGCAATTTTAACTTGCTCTGGAA**

**CEV genogroup I CATCAATCTGGATTCCTTTCCAGAACATAACATTTGCAATTTTAACTTGCTCTGGAA**

********** ****************** ***************************
