# Supplementary material for: Experimental infections of different carp strains with the carp edema virus (CEV) give insights into the infection biology of the virus and indicate possible solutions to problems caused by koi sleepy disease (KSD) in carp aquaculture
Source: Vet Res. 2017 Feb 21;48:12. doi: 10.1186/s13567-017-0416-7 (PMC5320791; doi:10.1186/s13567-017-0416-7)
Supplement: Supplementary file 2 — Additional file 2. Phylogenetic tree based on DNA sequences encoding for a 373 bp fragment of the P4a core protein of carp edema virus. Sequences published by Matras et al. [15] were obtained from GenBank. The positions of the sequences obtained from viruses used for the cohabitation experiments are indicated with arrows. Analysis was performed with PhyML 3.0 software based on the maximum-likelihood principle while tree rendering was performed with TreeDyn 198.3. [file 13567_2017_416_MOESM2_ESM.docx]

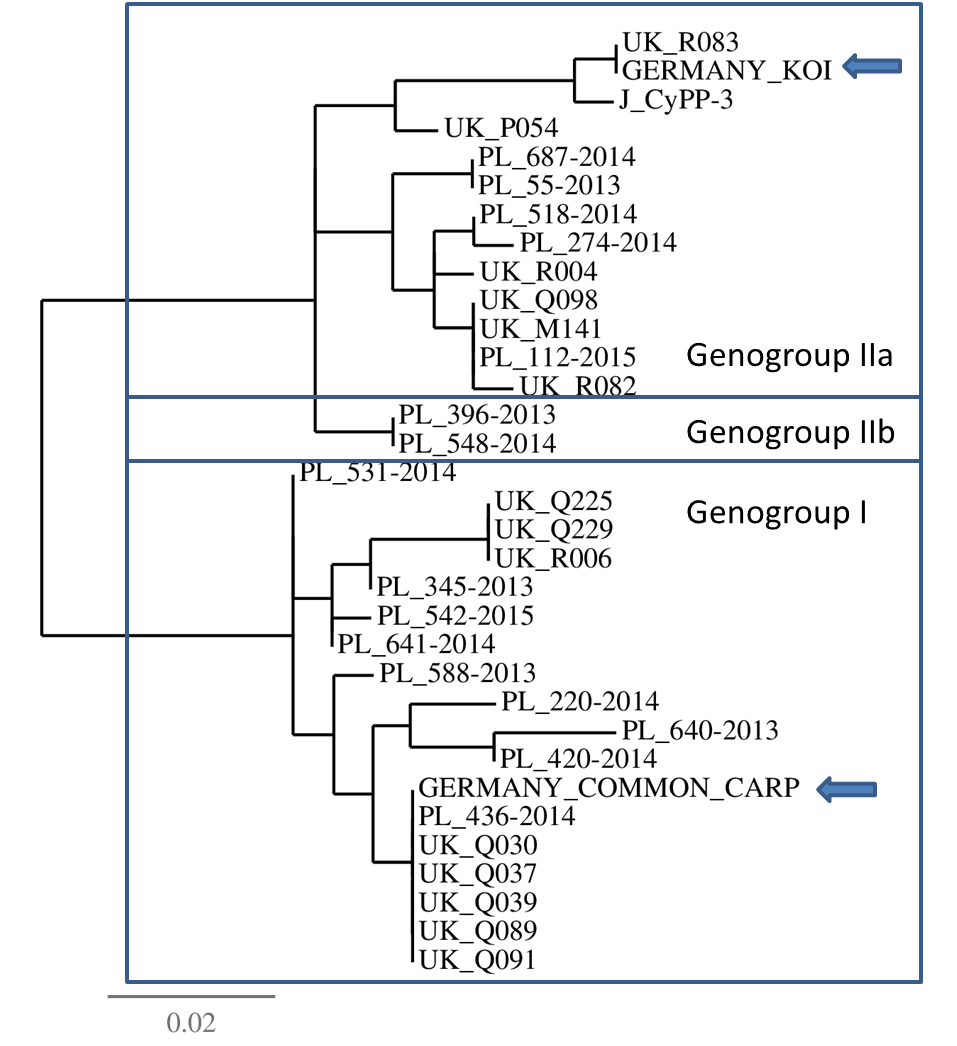


The scale bar indicates substitutions per nucleotide.

Genbank ID: PL_518-2014 (KX253997); PL_220-2014 (KX253998); PL_420-2014 (KX253999); PL_687-2014 (KX254000); PL_112-2015 (KX254001); PL_542-2015 (KX254002); PL_274-2014 (KX254003); PL_55-2013 (KX254004); PL_396-2013 (KX254005); PL_548-2014 (KX254006); PL_531-2014 (KX254007); PL_588-2013(KX254008); PL_436-2014 (KX254009); PL_641-2014 (KX254010); PL_345-2013 (KX254011); PL_640-2013(KX254012); UK_Q030 (KX254013); UK_Q037 (KX254014); UK_Q039 (KX254015); UK_Q089 (KX254016); UK_Q091 (KX254017); UK_Q225 (KX254018); UK_Q229 (KX254019); UK_R006 (KX254020), J_CyPP-3 (KX254021); UK_M141 (KX254022); UK_P054 (KX254023); UK_Q098 (KX254024); UK_R004 (KX254025); UK_R082 (KX254026); UK_R083 (KX254027).
