# Supplementary material for: Experimental infections of different carp strains with the carp edema virus (CEV) give insights into the infection biology of the virus and indicate possible solutions to problems caused by koi sleepy disease (KSD) in carp aquaculture
Source: Vet Res. 2017 Feb 21;48:12. doi: 10.1186/s13567-017-0416-7 (PMC5320791; doi:10.1186/s13567-017-0416-7)
Supplement: Supplementary file 3 — Additional file 3. Sequences of primers used in this study. Primers marked with “L” were used in qPCR for an estimation of virus load, primers marked with “E” were used in RT-qPCR expression analyses, primers marked with “P” were used for the amplification of gene fragments for plasmid based quantification. Primers marked with “S” were used for the amplification and sequencing of P4a gene fragment of CEV donor fish. [file 13567_2017_416_MOESM3_ESM.docx]

| **Gene** | **Primer/probe** | **Sequence 5’-3’** | **GenBank ID** | **Use** |
| --- | --- | --- | --- | --- |
| *CEV gene* |  |  |  |  |
| P4a (genogroup I) | CEVcarp_Sybr_qF1 | CATTTCCTAGTTTGTATGGCAAG |  | P,E,L |
|  | CEVcarp_Sybr_qR1 | TGATGATTGGAATAAGATGTCTGTC |  |  |
|  |  |  |  |  |
| P4a (genogroup IIa) | CEV_qF1 | TTTAGGAGGACAAGTAAAGTTACCA | KM283182 | E,L |
|  | CEV_qR1 | GCAAGTTATTTCGATGCCAAC C |  |  |
|  | CEV_probe1 | FAM-CCAGCTCCTACAAGGAAAGCAATTGA-BHQ |  | L |
|  |  |  |  |  |
| P4a (genogroups | CEFAS_CEVForB | ATGGAGTATCCAAAGTACTTAG |  | S |
| I and IIa) | CEFAS_CEVRevJ | CTCTTCACTATTGTGACTTTG |  |  |
|  |  |  |  |  |
| *CyHV-3 gene* |  |  |  |  |
| ORF89 | KHV-86F | GACGCCGGAGACCTTGTG | AF411803 | P,L |
|  | KHV-163R | CGGGTTCTTATTTTTGTCCTTGTT |  |  |
|  | KHV-109P | FAM-CTTCCTCTGCTCGGCGAGCACG-BHQ |  | L |
|  |  |  |  |  |
| *SVCV gene* |  |  |  |  |
| Glycoprotein | SVCV_G_qF | GCTACATCGCATTCCTTTTGC | Z37505.1 | P,L |
|  | SVCV_G_qR | GCTGAATTACAGGTTGCCATGAT |  |  |
|  |  |  |  |  |
| *Common carp genes* |  |  |  |  |
| 40S ribosomal protein S11 | q40S.FW1 | CCGTGGGTGACATCGTTACA | AB012087 | P,E |
|  | q40S.RV1 | TCAGGACATTGAACCTCACTGTCT |  |  |
|  |  |  |  |  |
| Interferon a2 | IFN_I_2_gsp_F1 | GAAACAAACTCAAATGTGGACATA | AB376667 | P |
|  | IFN_I_1_S_gsp_R1 | ACTCTTTCCAGGGACTTGTTTGCG |  |  |
|  | IFN_I_2_gsp_qF2 | GATGAAGGTGCCATTTCCAAG |  | E |
|  | IFN_I_2_gsp_qR3 | CACTGTCGTTAGGTTCCATTGCTC |  |  |
|  |  |  |  |  |
| Viperin | CycaVig1_F2 | CGCACCAGAGAGCAGAAAG | EX881775 and | P,E |
|  | CycaVig1_R2 | CTCAATAGGCAGCACGAAC | EX880905 |  |
|  |  |  |  |  |
| RNA dependent protein kinase | Cyca_PKR_qF1 | CCAACATCGTCCGCTACTACTC | EX880666 | P,E |
|  | Cyca_PKR_qR1 | GCGTGTCTCCCTCACAAAG |  |  |
|  |  |  |  |  |
